# Transparent Polymer Blends of Poly(methyl methacrylate) and Poly(propylene glycol)

**DOI:** 10.3390/polym14112171

**Published:** 2022-05-27

**Authors:** Andrei A. Korigodskii, Artem E. Zhirnov, Alexander S. Kechekyan, Sergey B. Zezin

**Affiliations:** 1 Department of Chemistry, Lomonosov Moscow State University, Leninskie Gory 1-3, 119991 Moscow, Russia; jyrnoff@gmail.com (A.E.Z.); zezinsb@yandex.ru (S.B.Z.); 2 Enikolopov Institute of Synthetic Polymer Materials, Russian Academy of Sciences, Profsoyuznaya ul. 70, 117393 Moscow, Russia; kec-alexander@yandex.ru

**Keywords:** polymer blends, poly(methyl methacrylate), poly(propylene glycol), plasticization, polymer glass, compression testing, XRD, DMTA, PMMA, PPG

## Abstract

Polymer blends, obtained by polymerization of methyl methacrylate in the presence of poly(propylene glycol), are investigated. Poly(propylene glycol) acts as a plasticizer, significantly lowering poly(methyl methacrylate)’s glass transition temperature and decreasing its elasticity modulus and yield stress. The mixture of methyl methacrylate with poly(propylene glycol) is more stable than its mixture with currently used poly(ethylene glycol), which leads to more uniform distribution and higher possible content of the plasticizer. Unlike low molecular weight plasticizers, poly(propylene glycol) is less prone to migration and exudation during manufacturing process and in use, and has low toxicity. Dynamic mechanical thermal analysis, compression testing and X-ray diffraction were used to investigate how the properties of the material depend on the content and molecular weight of the poly(propylene glycol) in the polymer blend. It was shown that the dependence of the glass transition temperature of methyl methacrylate polymerized in the presence of poly(propylene glycol) on the molar fraction of propylene glycol units is linear, and poly(propylene glycol) with lower molecular weight affects properties of the material stronger than poly(propylene glycol) with higher molecular weight. Therefore, the addition of poly(propylene glycol) allows to control the properties of poly(methyl methacrylate) easily and within wide range.

## 1. Introduction

Poly(methyl methacrylate) (PMMA) is a synthetic polymer of methyl methacrylate, an amorphous thermoplastic, which is widely used because it shows high impact strength, is lightweight, shatter-resistant, and easy to process [1]. Its outstanding properties include high transparency, weather resistance and scratch resistance.

Plasticizers are added to glassy polymers to increase their plasticity and elasticity [2]. They allow to improve the material’s frost resistance and brittleness [3], impact strength and manufacturability [4], and directionally change its heat, electrophysical [5], dielectric [6] and other properties. Reducing the glass transition temperature with the introduction of a plasticizer allows to expand the temperature range of the rubbery state of the polymer [7], and lowering the pour point facilitates the processing of the composition [8] improves the dispersibility of the filler and other ingredients of the mixture [9].

Low molecular weight plasticizers are widely used to modify properties of PMMA [9,10], but they possess disadvantages such as the ability to leave the material during use, which limits material longevity [11] and its applicability in medicine.

To suppress plasticizer migration, high molecular weight plasticizers are often preferred to ones with low molecular weight [12,13,14] as the diffusion coefficient of a plasticizer decreases rapidly with increase in its molecular mass [15]. Soft-chain oligomers and polymers are often used as such high molecular weight plasticizers [16,17,18,19]. In recent years, the market share of the high molecular weight plasticizers has grown rapidly [20].

Poly(ethylene glycol) is used to plasticize PMMA [21,22,23,24,25,26] because it combines high biocompatibility and good retention properties. However, its compatibility with PMMA is limited [7,27], which limits its usability and the range of available properties of the resulting materials as plasticizer should not form a separate phase.

We decided to investigate the properties of poly(propylene glycol) (PPG) as a plasticizer for poly(methyl methacrylate). Due to the additional methyl group, it is more compatible with the PMMA matrix, while still exhibiting high biocompatibility and low toxicity [28,29]. Besides this, it is readily available, cheap and remains in the polymer system during processing.

As we will show, introducing poly(propylene glycol) in the synthesis of the PMMA-based materials allows to directionally modify their mechanical and thermomechanical properties.

In the course of this work, a number of samples were synthesized by conducting a radical polymerization of the methyl methacrylate in the MMA–PPG mixture. Three poly(propylene glycols) of the different molar masses were used (with $M_{n}$ of 446, 1010 and 2025) to compare their plasticizing ability. Samples with the content of poly(propylene glycol) of 2, 5, 10 and 30 wt.% was obtained.

The resulting materials were investigated by means of X-ray diffraction, dynamic mechanical thermal analysis to determine the glass transition temperature, and uniaxial compression mechanical analysis to measure elastic modulus, yield stress and yield strain. The goal was to investigate if poly(propylene glycol) exhibits significant plasticizing effect and to determine how such an effect depends on the content and molar mass of PPG.

## 2. Materials and Methods

### 2.1. Materials

Methyl methacrylate was supplied by Scientific Research Institute of Polymers (Dzerzhinsk, Russia). Poly(propylene glycols) of three different molecular weights were used: Aldrich #81350 ($M_{n}$ 446), Voranol P1010 ($M_{n}$ 1010) and Merck #12303 ($M_{n}$ 2025). Di-(tert-butyl-cyclohexyl)-peroxydicarbonate was manufactured by Akzo Nobel (Perkadox-16).

### 2.2. Synthesis

The synthesis was carried out by the bulk polymerization of a mixture of methyl methacrylate and poly(propylene glycol). The polymerization mixture was prepared in a 5 mL glass tubes of the 8 mm diameter using 0.5 wt.% of di-(tert-butyl-cyclohexyl)-peroxydicarbonate as a free radical initiator. Then the mixture was stirred and the tubes were closed with a plastic cap. The polymerization was carried out at a temperature of 30 °C. The tubes were placed in a water bath to ensure the removal of excess heat generated during the polymerization, which otherwise leads to boiling of the reaction mixture. The end of the polymerization was determined by the complete solidification of the polymerization mixture, which took about two days. Then the samples were annealed by the stepwise temperature increase up to 110 °C. After annealing, the samples were removed by breaking the tubes. The samples were divided into 14 mm long cylinders using Kamach II 1060 CNC laser cutting machine with Lasea F2 95W carbon dioxide laser with 10.6 µm wavelength using 7 mm/s cutting speed. The resulting diameter of the samples amounted to 8 mm.

### 2.3. X-ray Diffraction

A Seifert-FPM URD6 X-ray diffractometer was used for recording the diffractograms. Radiation was generated from a copper anode tube (Cu Kα 1.5419 Å) using a BSV-29 X-ray generator operated at 30 kV and 20 mA. The scanning speed was 2°/min. The samples were 1 mm thick round plates, cut from 8 mm diameter cylinders.

### 2.4. Dynamic Mechanical Thermal Analysis

DMTA curves were recorded on a NETZSCH TMA 402F1 thermal analyzer. The samples were heated at 2 °C/min from 30 to 150 °C using silicon carbide oven. The stress was applied in sinusoidal mode, with 0.5 N constant component and 0.1 N amplitude. The period of oscillations was 10 s. The glass transition temperatures were determined as the maxima of temperature dependence of the loss tangent.

### 2.5. Compression Testing

A Shimadzu Autograph AGS 20 precision universal tester was used for compression testing. The compression test was preferred over the more common tensile test because it reflects changes in the elasticity and flow of materials without requiring complex sample preparation. The samples were cylinders 14 mm high and 8 mm in diameter. The uniaxial compression was conducted at 5 mm/min rate at 24 °C until stress exceeded 10 kN.

## 3. Results

### 3.1. Synthesis

At the room temperature, all the obtained samples were in the glassy state. All the samples were optically transparent (a photograph is available in the supplementary materials, Figure S1), with the exception of the sample with 30 wt.% of PPG with $M_{n}$ of 2025, which was highly opalescent (Figure S2). It shows that with increase in the molar mass of poly(propylene glycol), its compatibility with PMMA matrix decreases, so a homogeneous single phase material with a high MW PPG can only be obtained at the lower concentrations of PPG. Poly(propylene glycols) of the lower molar masses do not exhibit this behavior. The polymerization was complete, with Raman spectra showing the absence of residual monomer (supplementary materials, Figure S4).

### 3.2. X-ray Diffraction

**Figure 1:**
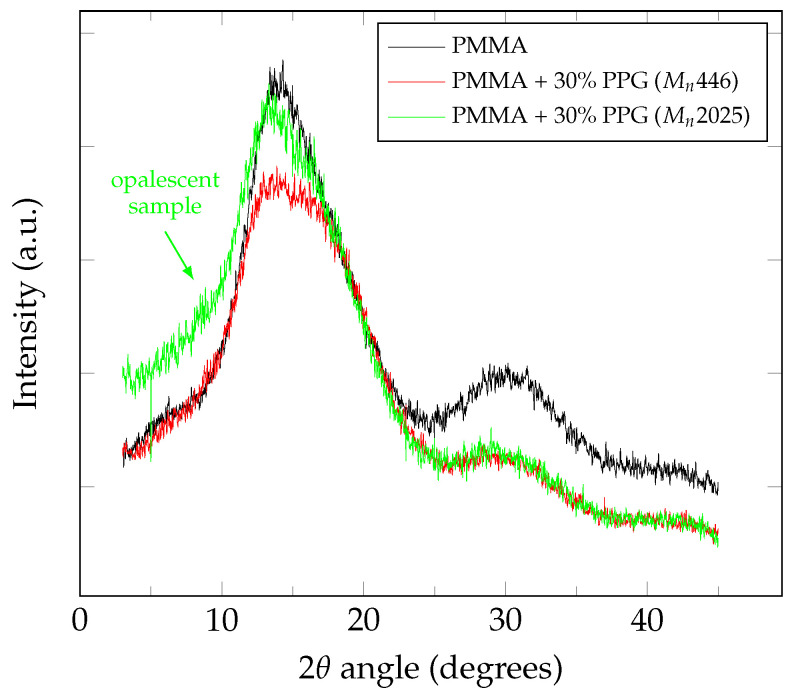
X-ray diffraction shows the difference between the low molecular weight PPG, which is compatible with PMMA at 30 wt.%, and the PPG of higher molecular weight, which causes opacity at such concentrations.

X-ray diffraction (XRD) was used to study the structural changes of the polymer during the plasticization (Figure 1). PMMA is known to be an amorphous polymer and shows three broad peaks at 2ϑ values of 12°, 30° and 42° [14]. The shape of the first most intense peak (diffuse halo) at 2ϑ value of 14° and d-spacing around 6.3 Å reflects the ordered packing of polymer chains, while the second peak at 2ϑ value of 30° and d-spacing of about 3.0 Å denotes the ordering inside the main chains [30].

### 3.3. Dynamic Mechanical Thermal Analysis

**Figure 2:**
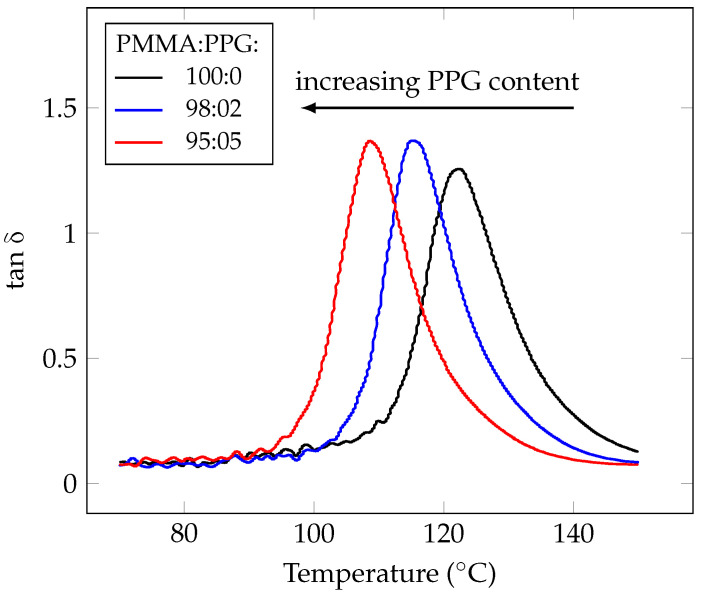
Dynamic mechanical thermal analysis of methyl methacrylate, polymerized in the presence of various content of poly(propylene glycol) ($M_{n}$ 446), showing the loss tangent as a function of temperature, is consistent with plasticization theory, wherein increasing plasticizer content corresponds to decreasing glass transition temperature of the resulting material.

**Figure 3:**
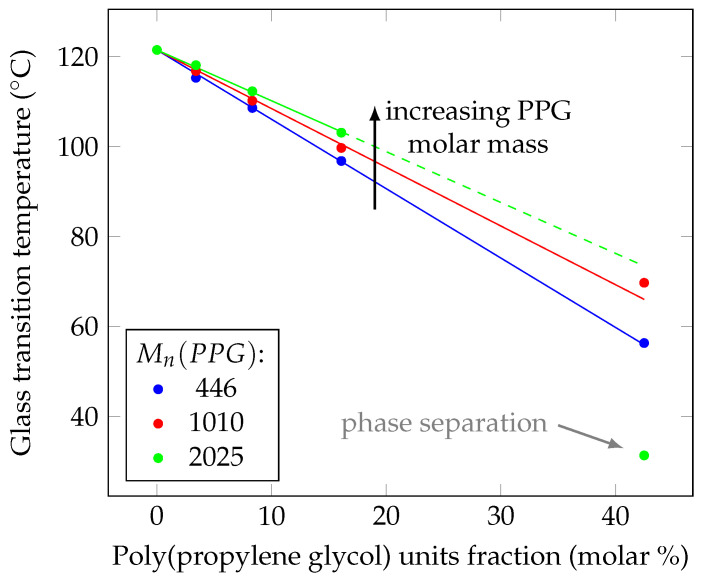
Effect of poly(propylene glycol) content on the glass transition temperature, measured with DMTA. Increasing molar fraction of PPG units in the polymer blend leads to linear decrease of the glass transition temperature. This effect weakens on the increase of PPG molar mass. The sample with 30 wt.% of PPG ($M_{n}$ 2025), which exhibits loss of transparency, is deviating strongly from the extrapolated dependency, unlike similar samples with PPG of lower molar mass.

Dynamic mechanical thermal analysis (Figure 2 and Figure 3) shows a steady decline in the glass transition temperature with increasing the content of poly(propylene glycol) in the blend. Poly(propylene glycol) with lower molecular weight reduces the glass transition temperature more than one with higher molecular weight.

### 3.4. Compression Testing

**Figure 4:**
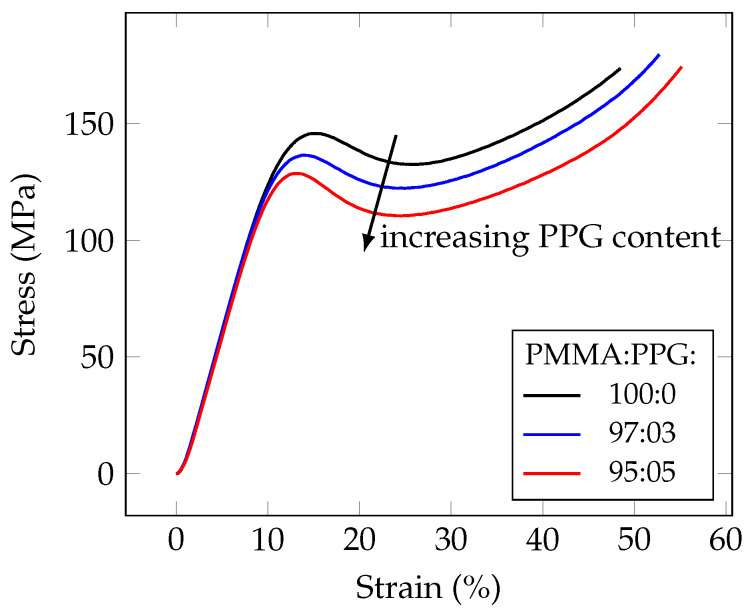
Comparison of the stress–strain responses during the uniaxial compression of PMMA, plasticized with various amounts of PPG $\left(M_{n}\;446\right)$, shows that increase in PPG content leads to decrease of yield strength.

The dynamometric measurements were conducted by the uniaxial compressing testing of cylinder-shaped samples, containing PPG with $M_{n}$ of 446. The typical resulting stress-strain curves are shown in Figure 4. The elastic moduli, values of the stress and strain at the yield point were calculated analyzing the stress-strain curves and plotted relating to the fraction of propylene glycol units in the polymer system. The ultimate strength was not analyzed, because, due to the plasticization, most of the samples did not collapse even at the maximum stress, which exceeded 300 MPa.

**Figure 5:**
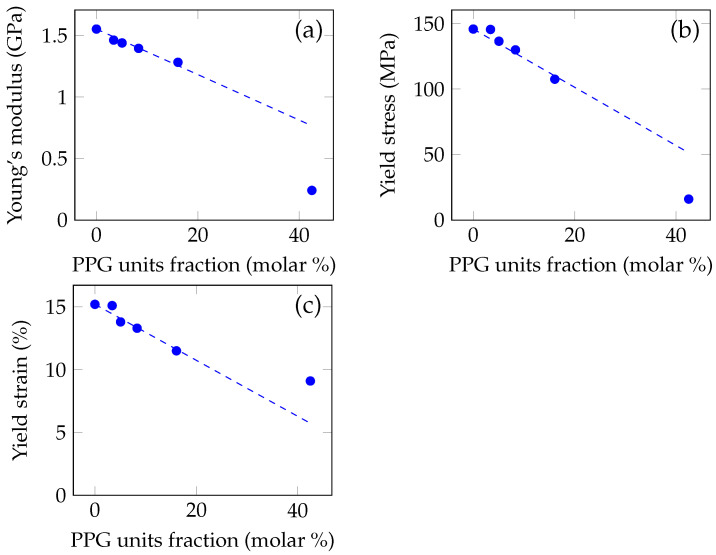
The results of the uniaxial compression testing illustrate the effect of the poly(propylene glycol) content on the mechanical properties. $M_{n}$ of PPG is 446. (**a**) The dependence of the elasticity modulus on the content of polypropylene glycol. (**b**) The dependence of the yield stress on the content of PPG. (**c**) The dependence of the yield strain on the content of PPG. The dependencies show that the modulus, the yield stress and strain monotonously decrease with increasing PPG molar fraction, and this relation is linear in the region of the moderate (up to 17%) fractions of the PPG units.

The compression testing shows a decline of the elastic modulus, yield stress and yield strain with increasing the poly(propylene glycol) content (Figure 5).

## 4. Discussion

### 4.1. X-ray Diffraction

Polymerization of methyl methacrylate in the presence of PPG with $M_{n}\;446$ leads to the formation of a compatible polymer blend which maintains optical transparency, while XRD pattern changes significantly relative to the pattern of pure PMMA: both peaks lose their intensity, while the first one changes shape, broadening (Figure 1). This can be understood as an evidence of decreasing homogeneity with introducing PPG to the polymer system. Addition of PPG with $M_{n}\;2025$, which results in loss of transparency, appears differently on the XRD pattern: while in area of larger ϑ it behaves the same as the sample with PPG ($M_{n}\;446$), the first peak almost repeats one of a pure PMMA, and the intensity of X-ray scattering at smaller angles (3–10°) increases. Moreover, XRD patterns confirm that the loss of transparency on addition of 30 wt.% of PPG ($M_{n}\;2025$) is not related to crystallization, and the material remains amorphous.

### 4.2. Dynamic Mechanical Thermal Analysis

A decrease in the glass transition temperature with an increase in the content of poly(propylene glycol) in the blend (Figure 3) confirms that it acts as a plasticizer.

Initially, the relationship between the content of the poly(propylene glycol) and the glass transition temperature of the resulting material were investigated using the mass fraction of PPG in the blend. In order to select a model that best fits this relationship, dependencies were calculated using molar fraction of the PPG units in the blend (according to the molar fraction rule) and the molar ratio of the PPG vs. PMMA units (according to the Zhurkov rule) [2].

It was found that experimental data are best described by the molar fractions rule, suggested by Kargin and Malinsky:

(1) $$\delta T_{g}=kx,$$

where *x* is the molar fraction of the propylene glycol units of all the polymer units.

The molar fraction of propylene glycol (PG) units was calculated according to the expression:

(2) $$x=\frac{w_{PPG}/M_{PGunit}}{\left(1-w_{PPG}/M_{MMAunit}\right)+w_{PPG}/M_{PGunit}},$$

where $w_{PPG}$ is the mass fraction of poly(propylene glycol).

Thus, the dependence of the glass transition temperature on the molar fraction of propylene glycol units in the mixture is linear, which allows to predict the glass transition temperature of poly(methyl methacrylate) with the addition of certain amounts of poly(propylene glycol) and design materials with the necessary $T_{g}$.

Results in Figure 3 show that the plasticizing effect (lowering the glass transition temperature) of poly(propylene glycol), distributed in the matrix of PMMA, decreases with an increase in its molecular weight. The slope of the approximating line can be interpreted as the efficiency of the plasticizer.

Decrease of the efficiency of poly(propylene glycol) as the plasticizer with increasing its molecular weight can be explained as follows.

First, the mobility of propylene glycol units is limited by the flexibility of the chain, and the units located in the middle of the poly(propylene glycol) molecule are less mobile than the units located at the ends of the molecule. With an increase in the molecular weight, the fraction of the units with limited mobility increases.

Second, this effect is associated with the indirect interaction of polymer molecules through a molecule of the plasticizer. A molecule of a low molecular weight plasticizer interacts with one atom or atomic group of a polymer molecule, shielding polymer–polymer interactions. A molecule of a high molecular weight plasticizer, such as poly(propylene glycol), forms multiple non-covalent bonds with polymer molecules, and polymer–plasticizer bonds are replacing some of the polymer–polymer bonds. Since one plasticizer molecule forms similar bonds with several groups of polymer molecules, they are interconnected through a plasticizer molecule. This leads to a decrease in the plasticizing effect compared with a low molecular weight plasticizer, and the larger the plasticizer molecule, the more such bonds are formed and the more the plasticizing effect decreases.

### 4.3. Compression Testing

As shown in Figure 5, the elastic modulus, as well as the stress and the strain at the yield point, decrease with increasing the poly(propylene glycol) content. This is due to the fact that molecules of poly(propylene glycol) in the polymer system weaken the interaction between PMMA chains and loosen the non-covalent bonds between them, effectively shielding them from each other to a degree proportional to the PPG content.

The dependence of the modulus, yield stress and yield strain on the unit fraction of poly(propylene glycol) is close to linear at the moderate concentrations of PPG (up to 10 wt.%). At concentration of 30 wt.%, when the content of the propylene glycol units in the polymer blend becomes comparable with one of methyl methacrylate units, the mechanical properties deviate strongly from the linear extrapolations: the elastic modulus and the yield stress are strongly reduced and the yield strain is increased. It can be explained by the change of character of the polymer–polymer interactions: the number of PMMA–PMMA interactions decreases drastically and the properties are defined more by the ones of poly(propylene glycol), which has incomparably smaller molar mass.

**Figure 6:**
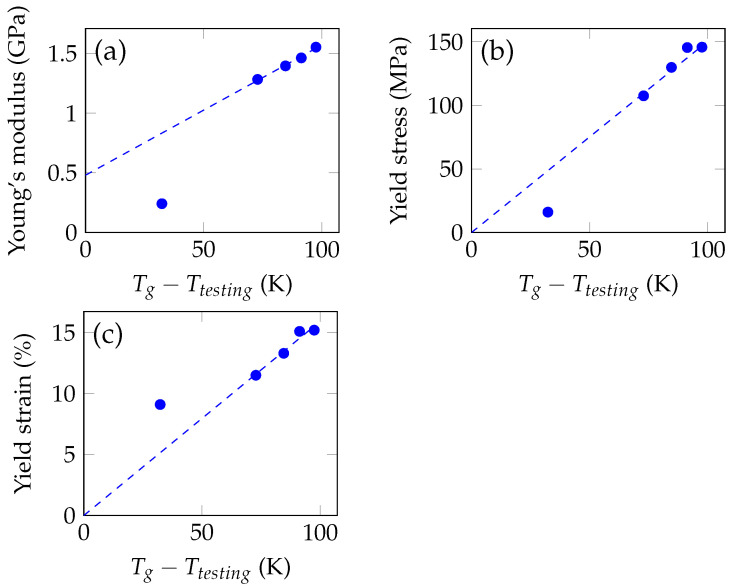
The results of the uniaxial compression testing were related to the difference of glass transition temperature and the testing temperature. $M_{n}$ of PPG is 446. (**a**) The dependence of the elasticity modulus on the relative glass transition temperature. (**b**) The dependence of the yield stress on the relative glass transition temperature. (**c**) The dependence of the yield strain on the relative glass transition temperature. The dependencies show linear relation between the mechanical properties and the relative glass transition temperature in the region of the moderate (up to 17%) fractions of the PPG units. Dependencies of yield stress and strain go through zero, unlike the elasticity modulus, which is expected, as at glass transition temperature the segmental mobility of macromolecules becomes unfrozen.

The results of the compression testing were also presented in Figure 6 as a relation of the mechanical properties (elasticity modulus, stress and strain at the yield point) to the relative glass transition temperature—a difference between the experimental (room) temperature and the glass transition temperature. The dependencies have remained close to linear. This illustrates the interchangeability, in some way, of plasticization and heating: the yield stress decreases with increasing plasticizer content in the same way it would decrease with heating, so the changes in the mechanical properties can be seen as consequences of a decrease in the glass transition temperature.

The dependencies of the yield stress and strain on the relative glass transition temperature pass through zero, unlike the dependence of the elastic modulus, because, after heating to the glass transition temperature, a polymer passes into the rubbery state, and the yield concept is not applicable anymore as the segmental mobility is already unfrozen even without external load.

## 5. Conclusions

Therefore, it was shown that poly(propylene glycol) can act as a suitable plasticizer for poly(methyl methacrylate). It was found that the possibility of obtaining homogeneous materials during the polymerization of methyl methacrylate with poly(propylene glycol) present is possible with a content of the latter up to 30 wt.% and that increasing the poly(propylene glycol) content leads to decrease of the glass transition temperature, elastic modulus, yield strain and yield stress. It was shown that the dependence of the glass transition temperature of the PMMA–PPG blend on the molar fraction of propylene glycol units is linear, and poly(propylene glycol) with lower molar mass affects properties of material stronger than poly(propylene glycol) with higher molar mass.

## Data Availability

Data in tabular form is available in the Supplementary Materials.

## Supplementary Materials

The following supporting information can be downloaded at: https://www.mdpi.com/article/10.3390/polym14112171/s1. The samples composition, IR absorbance and Raman spectroscopy methods and results, DMTA and compression testing results in tabular form, X-ray diffractograms and images of samples.

## Abbreviations

The following abbreviations are used in this manuscript:

PMMA	Poly(methyl methacrylate)
PPG	Poly(propylene glycol)
PG	Propylene glycol
MW	Molecular weight
XRD	X-ray diffraction
DMTA	Dynamic mechanical thermal analysis
