# Supplementary material for: Transparent Polymer Blends of Poly(methyl methacrylate) and Poly(propylene glycol)
Source: Polymers (Basel). 2022 May 27;14(11):2171. doi: 10.3390/polym14112171 (PMC9183039; doi:10.3390/polym14112171)
Supplement: Supplementary file 1 [file polymers-14-02171-s001.zip › polymers-1721166-supplementary.pdf]

Supplementary Materials: Transparent Polymer Blends of Poly(methyl methacrylate) and Poly(propylene glycol)

Andrei A. Korigodskii [1*], Artem E. Zhirnov [1], Alexander S. Kechekyan [2] and Sergey B. Zezin [1]

Synthesis

Materials of the compositions listed in Table S1 were obtained.

Table S1. Samples composition

Monomer	Plasticizing agent (P.A.)	M_n of P.A.	P.A. content, wt%
MMA	–	–	0
MMA	Poly(propylene glycol)	446	2; 3; 5; 10; 30
MMA	Poly(propylene glycol)	1010	2; 5; 10; 30
MMA	Poly(propylene glycol)	2025	2; 5; 10; 30

All the samples excluding the one containing 30 wt.% of PPG (M_n 2025) maintained optical transparency.

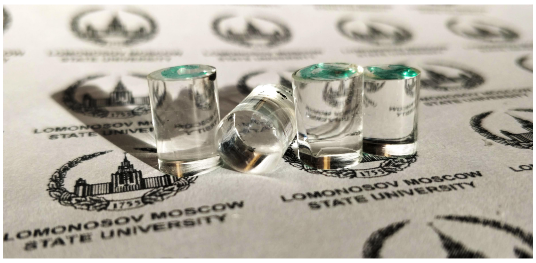

Figure S1. Typical samples of PMMA–PPG blend. All the obtained samples (except the one with 30 wt.% of PPG M_n 2025) are completely transparent.

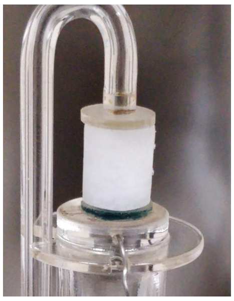

Figure S2. Sample with 30 wt.% of PPG (M_n 2025) is strongly opalescent.

Raman spectroscopy

Raman spectroscopy was conducted using Horiba Jobin-Yvon LabRam HR800 spectrometer with 488 nm laser. The cylinder samples of PMMA–PPG blends with different content of PPG (M_n446) were investigated. Methyl(methacrylate) and poly(propylene glycol) (M_n446) were in liquid form.

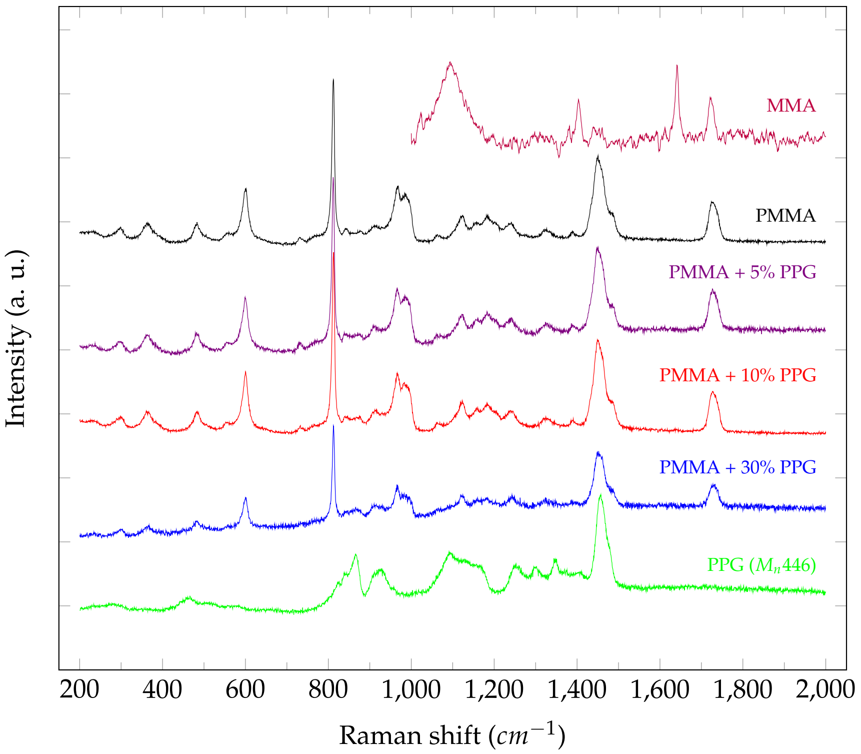

Figure S3. Raman spectra of the blends of poly(methyl methacrylate) and poly(propylene glycol) with M_n of 446.

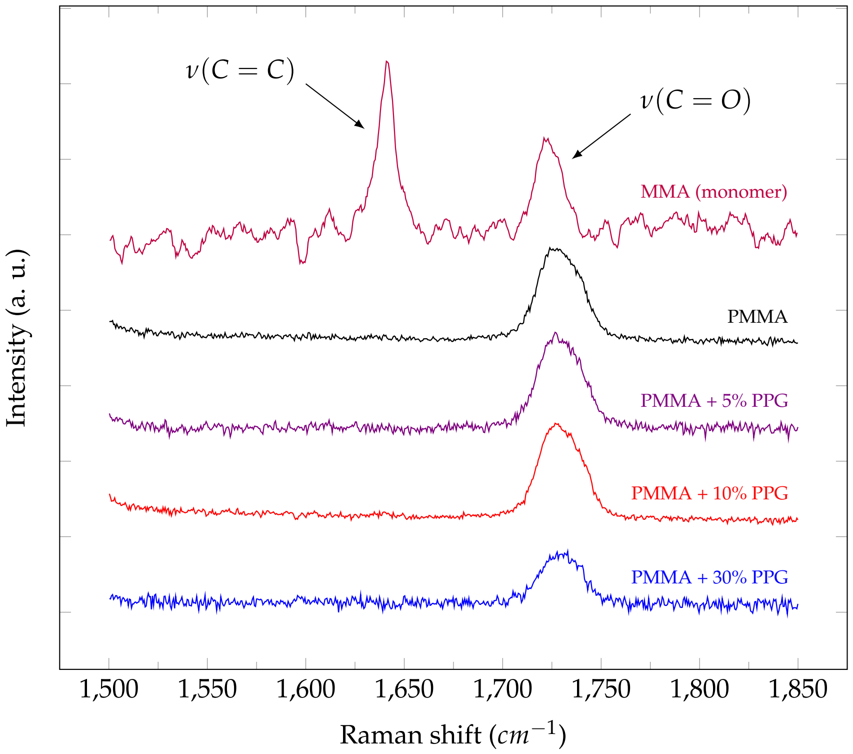

Figure S4. Raman spectroscopy confirms the absence of the monomer in the samples.

Infrared absorbance spectroscopy

A Specord M80 IR absorbance spectrometer was used to measure IR absorbance in 500–2000 cm^{-1} range with resolution of 4 cm^{-1}/pt. About 5 mg of the samples was filed with a needle file, rubbed in a mortar with KBr and then pressed into a disk (tablet) with a pneumatic press. Poly(propylene glycol) was investigated in the liquid form.

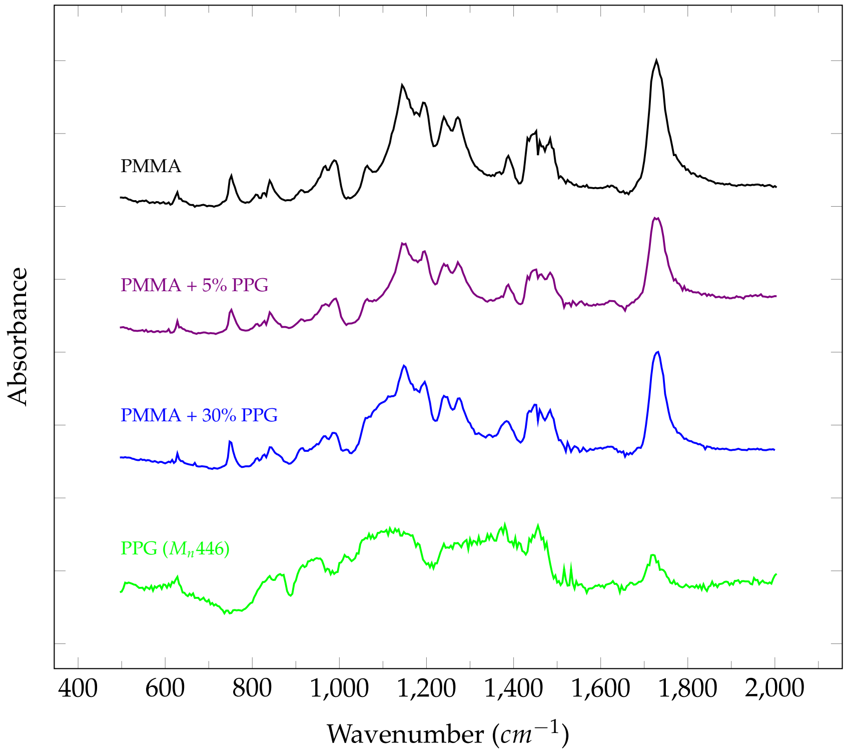

Figure S5. Infrared absorbance spectra of the blends of poly(methyl methacrylate) and poly(propylene glycol) with M_n of 446.

Dynamic mechanical thermal analysis

Table S2. DMTA tabular results

PPG units fraction, %	PPG M_n 446 T_g, °C	PPG M_n 1010 T_g, °C	PPG M_n 2025 T_g, °C
0.0	121.5	121.5	121.5
3.4	115.3	116.8	118.1
8.3	108.6	110.2	112.3
16.1	96.8	99.7	103.1
42.5	56.3	69.7	31.3

Compression testing

Table S3. Compression testing tabular results

PPG content, wt.%	PPG units fraction, %	Elastic modulus, GPa	Yield strength, MPa	Yield strain, %
0	0	1.55	145.8	15.2
2	3.4	1.46	145.5	15.1
3	5.1	1.44	136.5	13.8
5	8.3	1.40	129.9	13.3
10	16.1	1.28	107.5	11.5
30	42.5	0.24	16.0	9.1

X-ray diffraction

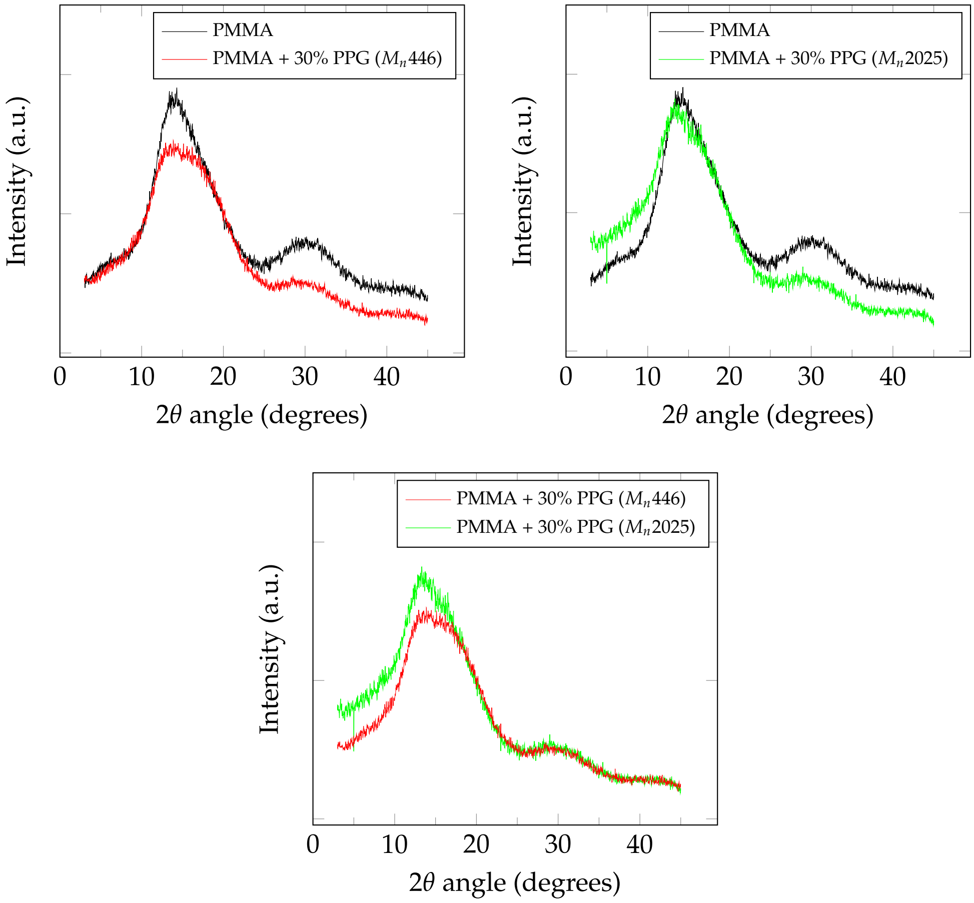

Figure S6. X-ray diffraction. The samples of pure PMMA and PMMA with 30 wt.% of PPG $(M_n 446)$ are transparent, while the sample of PMMA with 30 wt.% of PPG $(M_n 2025)$ is opalescent.
